# Maintaining prostate contouring consistency following an educational intervention

**DOI:** 10.1002/jmrs.168

**Published:** 2016-03-16

**Authors:** Luke Nicholls, Peter Gorayski, Michael Poulsen, Ashley W. Plank, Karlissa Schick, Thuy Pham, Eric L. H. Khoo

**Affiliations:** ^1^ Radiation Oncology CentresCairnsQueenslandAustralia; ^2^ School of MedicineUniversity of QueenslandSt. LuciaQueenslandAustralia; ^3^ Radiation Oncology CentresSpringfieldQueenslandAustralia; ^4^ Radiation Oncology CentresSt Andrew's Cancer Care CentreToowoombaQueenslandAustralia; ^5^ Oncology Research AustraliaSt Andrew's HospitalToowoombaQueenslandAustralia

**Keywords:** Contouring, education intervention, inter‐observer variation, prostate, target volume delineation

## Abstract

**Introduction:**

The aim of this study was to assess variation in prostate contouring 12 months following a structured interactive educational intervention (EI) and to test the hypothesis that EIs positively impact on prostate contouring accuracy and consistency long term.

**Methods:**

A common set of computed tomography (CT) and magnetic resonance imaging (MRI) data sets were used to assess prostate contouring consistency before, immediately after and 12 months following an EI. No further EIs were provided after the initial EI. Contour variation was assessed using the volume ratio (VR), defined as the ratio of the encompassing volume to common volume.

**Results:**

Of the original five radiation oncologists (ROs) at baseline, four completed all assessments, and one was unavailable at 12 months follow‐up. At 12 months, mean VR deteriorated by 3.2% on CT and 1.9% on MRI compared to immediately post EI. Overall, compared to the pre‐EI baseline VR, an improvement of 11.4% and 10.8% was demonstrated on CT and MRI, respectively.

**Conclusion:**

Good retention of applied knowledge 12 months following an EI on prostate contouring was demonstrated. This study advocates for EIs to be included as part of continuing medical education to reduce contour variation among ROs and improve knowledge retention long term.

## Introduction

Recent advances in the delivery of radiation therapy (RT) for clinically localised prostate cancer have enabled dose escalation and greater sparing of organs at risk (OAR).^1^, ^2^ To minimise radiation‐induced toxicity to the rectum and bladder neck, co‐registration of magnetic resonance imaging (MRI) with planning computed tomography (CT) data sets has been incorporated into clinical practice to improve soft tissue delineation.^3^, ^4^, ^5^

Due to normal anatomical variations, physiological movement (internal motion) and inherent uncertainties in patient positioning (setup error), RT to the prostate is subject to inter‐ and intra‐fraction variation. Planning target volumes (PTV) are thus generated to account for this uncertainty. Image‐guided RT using intra‐prostatic fiducial markers can reduce inter‐fractional treatment variation and PTV margins^6^ while specialised systems (e.g., Calypso^®^ extra‐cranial tracking) can monitor prostate motion during treatment to improve accuracy.^7^

Contouring variability is a major source of error in RT delivery, having an impact on treatment accuracy similar to organ motion and setup variation.^8^ Therefore, quality assurance of target volume (TV) delineation among radiation oncologists (ROs) is essential to improve consistency. The co‐registration of CT and MRI has been shown to improve clinical target volume (CTV) delineation and reduce inter‐observer variability^9^ while dedicated anatomical and contouring education interventions (EI) have been trialled.^10^

This study examines the longer term impact of an education initiative (EI) to improve TV delineation by ROs 12 months post EI. It is hypothesised that an EI would positively impact prostate contouring in the long term. This is a follow‐up study to that reported by Khoo et al.^11^

## Methods and Materials

### Contouring

This study is predominantly a quality assurance audit and thus a request for waiver of Human Research Ethics Committee review was approved by Oncology Research Australia. The same three CT and MRI data sets used in a previous report^11^ were again used for contouring and consisted of three clinical scenarios: a patient with a small prostate (42.5 cm^3^), large prostate (66.4 cm^3^) and a right hip prosthesis. Four out of the five ROs from the previous study contoured the entire prostate gland for each patient on CT and MRI images. One participating RO (‘RO4’) was unable to contribute due to work relocation.

No further EIs were provided. The initial EI occurred 12 months prior and consisted of three formal components. These consisted of a series of anatomy lectures, completion of contouring modules using *Prost‐a‐doodle* software^12^ and peer‐review of contoured volumes. For each patient, the prostate was contoured on the planning CT data set, then on the MRI data set, without referral back to the just completed contour on the CT data set. The study schedule used in the EI is summarised in Table 1.

**Table 1:** Study schedule during the EI and subsequent follow‐up

Schedule	Events	Duration of each session	Study
Month 1	Contouring on CT data set first, then MRI data set consecutively for 3 patients	60–90 min	Khoo et al.^11^
Month 2	As per month 1	60–90 min	
EI (1 session per week over 3 weeks)	Session 1: MRI prostate anatomy session Session 2: MRI prostate anatomy session Session 3: Practical session	60 min each	
Month 3	As per month 1	60–90 min	
Month 4	As per month 1	60–90 min	
Month 12	As per month 1	60–90 min	Current study

CT, computed tomography; MRI, magnetic resonance imaging; EI, education intervention.

The Philips Brilliance 16‐slice CT scanner (Philips, Cleveland, OH) with 1‐mm slices and the GE Healthcare 1.5T, Excite Platform, eight‐channel coil MRI scanner (GE Healthcare, Milwaukee, WI) with T2‐weighted sequence with 2 mm slices were used. All data sets were imported into the Eclipse treatment planning system version 8.9 (Varian Medical Systems, Palo Alto, CA). Coregistrations of CT and MRI data sets were based on prostate fiducial markers. Neither the ROs previous contours nor their colleagues’ contours were available during any contouring session.

### Analysis

**Figure 1:**
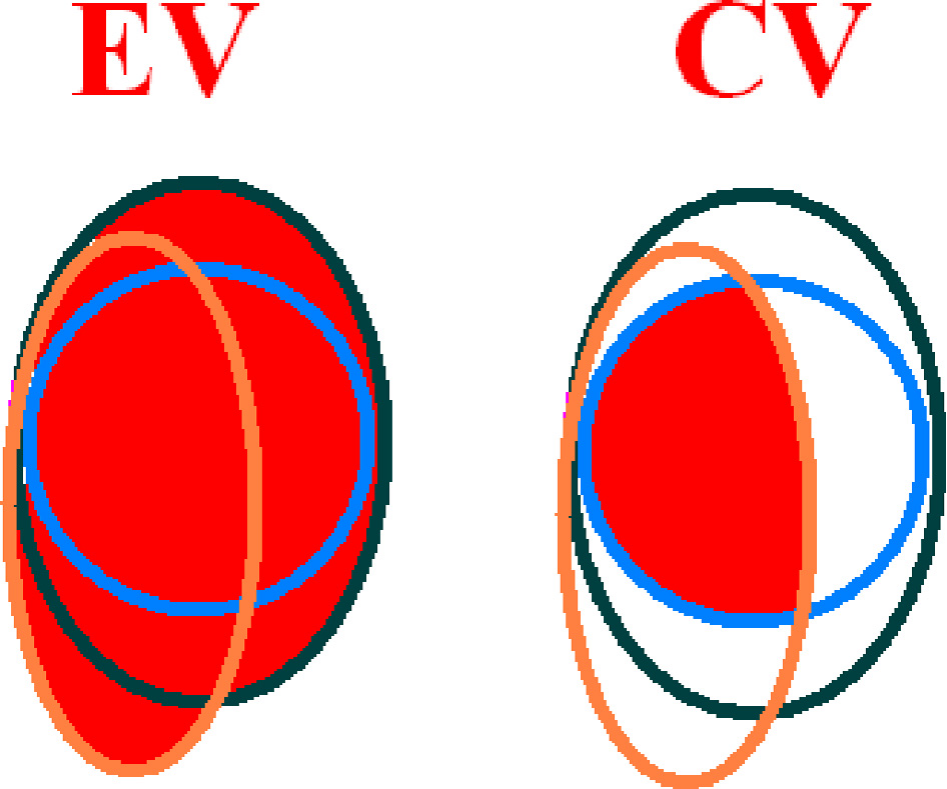
Volume ratio (VR) = encompassing volume (EV)/common volume (CV).

Observer variation was measured as the ratio of encompassing volume (EV) (the volume of the union of a set of TVs) to common volume (CV, the volume of the intersection of the same set of TVs)^3^ (See Fig. 1). This metric is referred to as volume ratio (VR) and has also been referred to as the Concordance Index in other contouring analyses.^10^ For a set of identical TVs, VR is 1, indicating no observer variation. As observer variation increases, VR increases.

To determine intra‐observer variation, the VR was calculated for each RO, patient and imaging modality from the TVs as contoured by the RO in each of the 2 months before, immediately following and 12 months following the EI (see Table 2). Boolean analysis was used to measure the degree of volume overlap for EV and CV. The contour variation was assessed based on changes in the VR. A comparison (percentage change) of VR before, immediately after and 12 months after the EI was determined. Due to the small sample size, descriptive statistics are presented without formal inference. Inter‐observer contour variation was not calculated.

**Table 2:** Individual and mean intra‐observer VR before, immediately after and 12 months following the EI for computed tomography and magnetic resonance imaging modalities

Parameter	Education	RO1	RO2	RO3	RO5	Mean VR
CT CTV	Before	1.44	1.43	1.99	1.32	1.55
	After	1.31	1.32	1.4	1.25	1.32
	12 months	1.26	1.31	1.66	1.25	1.37
MRI CTV	Before	1.19	1.18	1.46	1.46	1.32
	After	1.14	1.16	1.18	1.14	1.16
	12 months	1.16	1.12	1.26	1.18	1.18

VR, volume ratio; CT, computed tomography; CTV, clinical target volume; MRI, magnetic resonance imaging; EI, education intervention.

## Results

**Figure 2:**
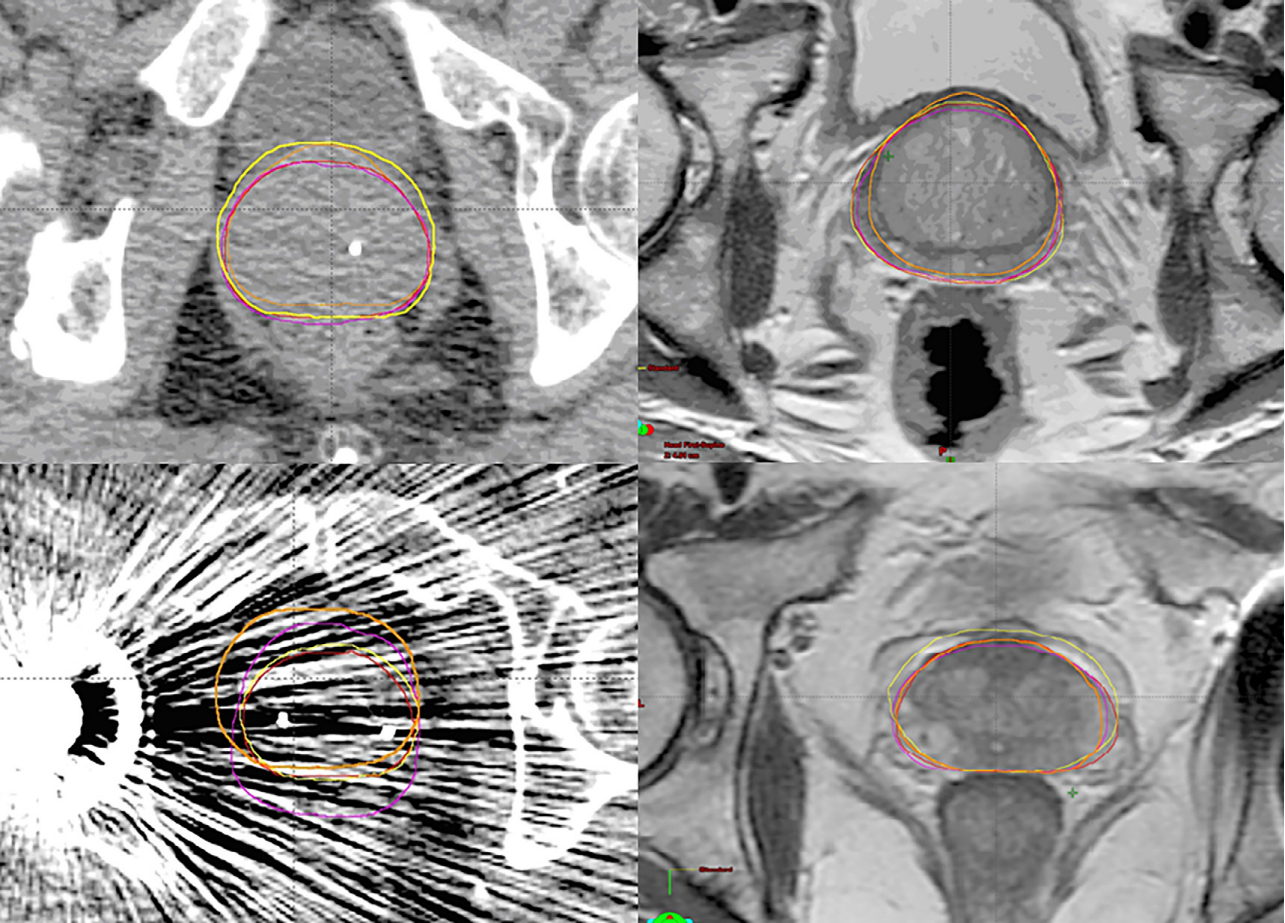
Prostate contouring at the mid‐gland level on computed tomography and magnetic resonance imaging demonstrating variability in target volume delineation between four radiation oncologists for patients 1 (above) and 3 (below).

Prostate contours at the mid‐gland level on CT and MRI images are outlined in Figure 2. The VR was stable for three ROs, however, VR regressed back to its level prior to the EI for one participant. The VR measurements were better for MRI contoured volumes compared to CT.

**Figure 3:**
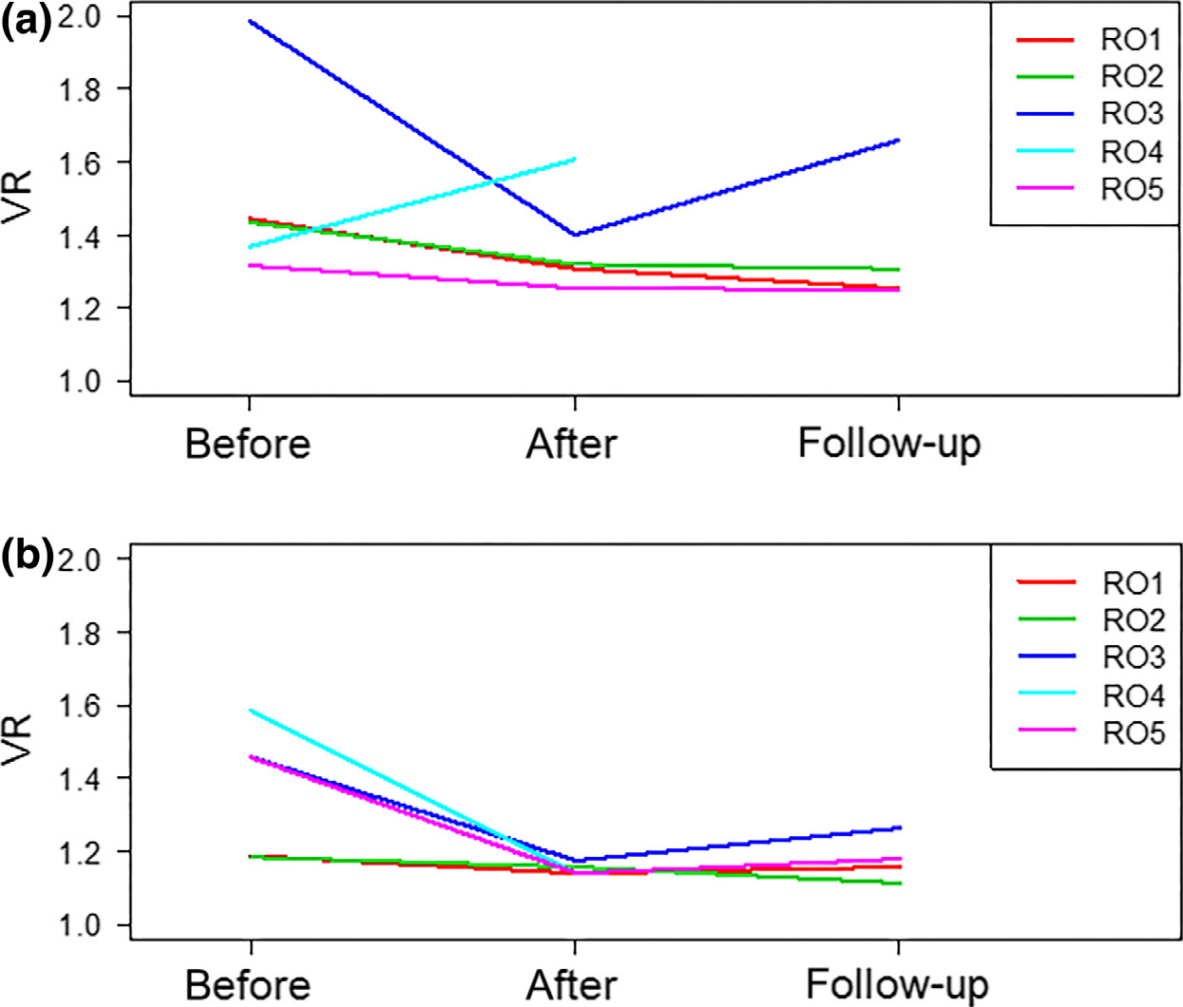
Mean intra‐observer volume ratio before, immediately after and 12 months following the education intervention for (A) computed tomography and (B) magnetic resonance imaging modalities.

There was a differential impact of the EI among ROs. For the CT data set, the mean VR of the 4 ROs after the EI and at 12 month follow‐up was 1.31, 1.32, 1.40, 1.25 and 1.26, 1.31, 1.66, 1.25, respectively. For the MRI data set, the mean VR after the EI and at 12 months was 1.14, 1.16, 1.18, 1.14 and 1.16, 1.12, 1.26, 1.18, respectively. The mean VRs for each RO for CT and MRI data sets and various follow‐up times are displayed in Table 2 and Figure 3.

**Figure 4:**
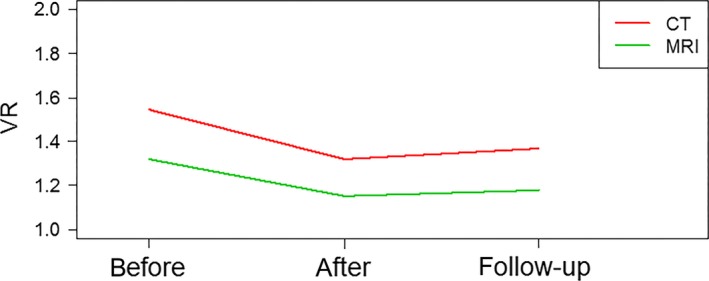
Mean intra‐observer volume ratio before, immediately after and 12 months following the education intervention averaged for all radiation oncologists and patients.

Mean VR measured after the EI compared to 12 month follow‐up for all ROs deteriorated by 3.2% (CT) and 1.9% (MRI) respectively. Overall, there is an improvement of 11.4% (CT) and 10.8% (MRI) from the baseline VR calculated prior to the EI. The mean intra‐observer VR averaged over all ROs and patients is shown in Figure 4 suggesting good retention over time.

## Discussion

Intra‐ and inter‐observer contouring variability is well described not only in localised prostate, but also for lung,^13^ head and neck^14^ and breast cancers.^15^ Despite no widely accepted method of systematic contour comparison, volume‐based metrics are the most frequently used assessment parameter.^10^ Our updated data 12 months following an EI to improve prostate contouring suggests that retention of knowledge is maintained over time.

To our knowledge, this is the first study to assess the long‐term impact of a structured EI on prostate contouring for ROs. This analysis focuses on knowledge and skill retention and has demonstrated an ongoing reduction in intra‐observer contour variation following a structured and interactive EI. While the mean VR 12 months after the EI deteriorated as a group compared to immediately after, the improvement from baseline was significant. This impact was greater on CT than MRI volumes.

This study contributes to the existing literature and provides additional insights into prostate contouring variability. Fiorino et al.^16^ demonstrated a 10–18% inter‐ and 5% intra‐observer variability in prostate volume contouring and Nakamura et al.^17^ exposed a wide variety of prostate volume definition among Japanese ROs. Gao et al.^18^ compared prostate gland delineations to images from the Visible Human Project and revealed that observers consistently underestimated the posterior portion of the prostate gland. This may have clinical implications as these observers may be inadvertently omitting the peripheral zone of the prostate, a region in which 70% of prostate adenocarcinoma develops.

Retention of acquired knowledge by health care professionals is a high priority across different disciplines. After 1 year, only 60–70% of knowledge is retained while skill sets appear to deteriorate more rapidly.^19^, ^20^ In our study, this inherent decay was most striking in RO3, who improved initially with the group, but subsequently returned to an earlier pattern of prostate contour over‐estimation. Various institutions and training bodies acknowledge this phenomena and mandate regular re‐training to maintain competency as repeated retrieval of information is crucial to long‐term retention. For example resuscitation councils worldwide traditionally recommend healthcare providers receive Advanced Life Support retraining or refresher course at least every 2 years.^20^ Recertification in RO contouring may be necessary to ensure quality compliance over time in an effort to reduce clinician error. Currently, no formal contouring recertification is required for radiation oncology fellows of the Royal Australian and New Zealand College of Radiologists (RANZCR).

Given the potential adverse clinical sequelae of CTV variation on patient outcomes, there has been a significant shift in contouring teaching and assessment. The American Society for Radiation Oncology (ASTRO) and the European Society for Radiotherapy and Oncology (ESTRO) both have established contouring programmes across multiple tumour streams. ASTRO have recently incorporated online contouring programmes in its annual meeting and are now presenting an ‘eContouring’ programme for radiation oncology trainees. Common among these programmes, participants can assess their own contour variability and compare their volumes to those of the instructor and their peers. The Canadian Association of Radiation Oncology (CARO) has recently trialled a contouring ‘boot camp’ with promising results.^21^ It has been shown that interactive workshops similar to those mentioned above result in significant changes in clinical practice as opposed to didactic sessions alone.^22^ There is currently no formal contouring training by the faculty of radiation oncology of RANZCR, however, close links with ESTRO are established.

Other means of improving contour variation include the use of published contouring atlases, institutional protocols, peer review of positron emission tomography (PET) or MRI image fusion and regular contouring quality assurance between radiation oncology departments. Intra‐ and inter‐observer variation in TV delineation has been shown to be reduced by implementing a departmental, national or international protocol.^23^, ^24^ The incorporation of a ‘refresher note’ into these protocols, a document outlining common contouring pitfalls and errors, may also play a role. The increasing practice of site specific weekly contouring quality assurance meetings in which RO contouring is peer reviewed prior to dosimetric planning appear to be a sound way forward in terms of increasing the consistency amongst ROs. Moreover, this provides a valuable educational forum for trainees.

The limitations of this single institution study include small sample size, loss of follow‐up of one original participating RO and the absence of a control or gold standard group. The absence of a gold standard contour makes it impossible to make conclusions about the absolute accuracy of contours. The choice of this gold standard or reference contour varies in the literature from a mathematical average contour, an RO‐ or radiologist‐defined contour, or a consensus contour that is decided upon by a panel of experts.^10^ The contour data sets from the previous study were lost due to an upgrade of the radiation treatment planning system in the department. However, our study used the same concept of VR methodology and the comparison between VR pre‐, post‐ and 12 months following the EI remains valid. Lastly, data on the frequency of prostate contouring for each of the ROs were not formally assessed. All ROs in this study regularly treat localised prostate cancer, however, absolute patient numbers treated were not available. Regular prostate contouring would appear to be the most robust way of consolidating contouring knowledge and may explain the variation amongst the ROs in the study.

Possible follow‐up studies in this domain could assess the significance between RO prostate contouring consistency and the number of localised prostate cancers treated per year. Furthermore, studies randomising ROs to an EI versus no EI, assessing contouring consistency in other tumour sites and assessing the required frequency of EIs to maximise knowledge retention over time may lead to further gains in contouring quality assurance.

## Conclusion

Contour variation impacts the accuracy of RT as significantly as organ motion and setup variability. Novel methods to improve contouring consistency should be rigorously pursued to match recent technological advancements in RT delivery. This contouring audit has shown that 12 months following a structured EI consisting of anatomy tutorials, contouring modules and peer review, adequate retention of knowledge and subsequent improvement in contouring consistency was demonstrated in a small cohort of ROs treating localised prostate cancer. The improvements in contour consistency achieved herein advocate for EIs to be included as part of continuing medical education for ROs treating localised prostate cancer. Further studies are required to further define the frequency with which such EI should be incorporated into clinical practice.

## Conflict of Interest

The authors declare no conflict of interest.
